# Tear Proteomics in Infants at Risk of Retinopathy of Prematurity: A Feasibility Study

**DOI:** 10.1167/tvst.13.5.1

**Published:** 2024-05-01

**Authors:** Chloe Shipton, Julie Aitken, Samuel Atkinson, Richard Burchmore, Ruth Hamilton, Helen Mactier, Suzanne McGill, Eoghan Millar, Anne Cees Houtman

**Affiliations:** 1 Royal Hospital for Children, Glasgow, Scotland, UK; 2 Princess Royal Maternity, Glasgow, Scotland, UK; 3 University of Aberdeen, School of Medicine, Medical Sciences and Nutrition, Foresterhill, Aberdeen, Scotland, UK; 4 University of Glasgow, Wolfson Wohl Cancer Research Centre, Bearsden, Glasgow, Scotland, UK; 5 Royal Hospital for Children, Glasgow, Glasgow, Scotland, UK; 6 University of Glasgow, Wolfson Wohl Cancer Research Centre, Bearsden, Glasgow, Scotland, UK

**Keywords:** retinopathy of prematurity (ROP), proteomics, screening, mass spectrometry (MS), biomarkers

## Abstract

**Purpose:**

This feasibility study investigated the practicability of collecting and analyzing tear proteins from preterm infants at risk of retinopathy of prematurity (ROP). We sought to identify any tear proteins which might be implicated in the pathophysiology of ROP as well as prognostic markers.

**Methods:**

Schirmer's test was used to obtain tear samples from premature babies, scheduled for ROP screening, after parental informed consent. Mass spectrometry was used for proteomic analysis.

**Results:**

Samples were collected from 12 infants, which were all adequate for protein analysis. Gestational age ranged from 25 + 6 to 31 + 1 weeks. Postnatal age at sampling ranged from 19 to 66 days. One infant developed self-limiting ROP. Seven hundred one proteins were identified; 261 proteins identified in the majority of tear samples, including several common tear proteins, were used for analyses. Increased risk of ROP as determined by the postnatal growth ROP (G-ROP) criteria was associated with an increase in lactate dehydrogenase B chain in tears. Older infants demonstrated increased concentration of immunoglobulin complexes within their tear samples and two sets of twins in the cohort showed exceptionally similar proteomes, supporting validity of the analysis.

**Conclusions:**

Tear sampling by Schirmer test strips and subsequent proteomic analysis by mass spectrometry in preterm infants is feasible. A larger study is required to investigate the potential use of tear proteomics in identification of ROP.

**Translational Relevance:**

Tear sampling and subsequent mass spectrometry in preterm infants is feasible. Investigation of the premature tear proteome may increase our understanding of retinal development and provide noninvasive biomarkers for identification of treatment-warranted ROP.

## Introduction

In retinopathy of prematurity (ROP), normal development of retinal blood vessels (angiogenesis) is disrupted. In the United Kingdom, infants born before 31 weeks’ gestation and/or those weighing less than 1501 g at birth are screened for ROP.^1^ Most cases resolve spontaneously but approximately 4% of at-risk infants develop sight-threatening disease which if untreated can lead to retinal detachment and blindness.^2^ Fourteen percent to 26% of adult patients with a history of ROP developed retinal detachments.^3^^,^^4^

Treatment aims to reduce proangiogenic factors and may include peripheral retinal ablation and/or intravitreal injection of antivascular endothelial growth factor (anti-VEGF) drugs.^2^ Screening of at-risk infants by indirect ophthalmoscopy and/or wide-field retinal imaging requires mydriasis, use of an eyelid speculum and scleral indentation, and may be accompanied by adverse effects, including cardiovascular instability.

ROP screening is usually performed by pediatric ophthalmologists. In the United Kingdom, three-quarters of consultant pediatric ophthalmology posts are unfilled.^5^ Although small relative to societal costs of blindness, the cost of screening for ROP is substantial.^6^ Lower cost alternatives or adjuncts are therefore desirable, especially for ROP screening in lower-income economies.

Tear fluid has a relatively high concentration of tear proteins^7^ and is readily accessible via minimally invasive methods. Tear proteomics have identified possible biomarkers, including growth factors and VEGF, in other ocular conditions.^8^^–^^10^ These proteins have multiple functions, including angiogenesis, immune or inflammatory response, visual perception, and metabolism.^7^ Schirmer's test is commonly used to assess aqueous tear deficiency^11^ by placing a strip of filter paper, in which tears are collected through capillary action, in the conjunctival fornix.

This TEAr pRoteomics Determine ROP Stage (TEARDROPS) feasibility study sought primarily to investigate the feasibility of collecting tears from preterm infants at risk of ROP and analyzing tear proteins which might have potential as prognostic markers for ROP. Infants produce less tears than adults,^12^^,^^13^ so this feasibility study addressed whether tear samples could be taken and whether proteins could be detected in the samples using proteomics with mass spectrometry (MS) used to detect hundreds of proteins within the tears. The three previous studies evaluating tears in this cohort have evaluated changes in up to four separate proteins.^14^^–^^16^ We also investigated whether any detected proteins might be associated with the development of ROP, which may enable more accessible screening methods.

## Methods

This prospective feasibility study was undertaken in the neonatal unit of Princess Royal Maternity, Glasgow, Scotland, United Kingdom. Eligible babies were those at risk of ROP according to UK national screening guidelines.^1^ Exclusion criteria included conjunctivitis or congenital ocular anomaly. The planned sample size was chosen as a convenience number for a preliminary, feasibility investigation. Eligible participants' parents or guardians were invited to participate and given an information leaflet. Time was allowed to consider the request and ask questions. Parents/guardians of all participating infants gave written consent to participate. The study was approved by NHS Health Research Authority, Yorkshire and The Humber - Leeds East Research Ethics Committee (REC reference: 21/YH/0214) and adheres to the principles of the Declaration of Helsinki.

Gestational age (GA), birth weight, postnatal age (PNA), and oxygen use at sample collection, daily weight gain from birth until sample collection, ethnicity, and ROP status were noted. Postnatal age was added to GA to calculate postmenstrual age (PMA) at sample collection. A postnatal growth ROP (G-ROP) score was calculated and used to stratify infants as high or low risk for ROP. The G-ROP scores expand 2 national screening criteria, with a lower GA of 28 weeks and a lower birth weight of 1051 g. It also incorporates three additional measurements of postnatal weight gain (<120 g 10–19 days PNA, <180 g 20–29 days PNA, and <170 g 30–39 days PNA) and presence or absence of hydrocephalus.^17^

**Figure 1.**
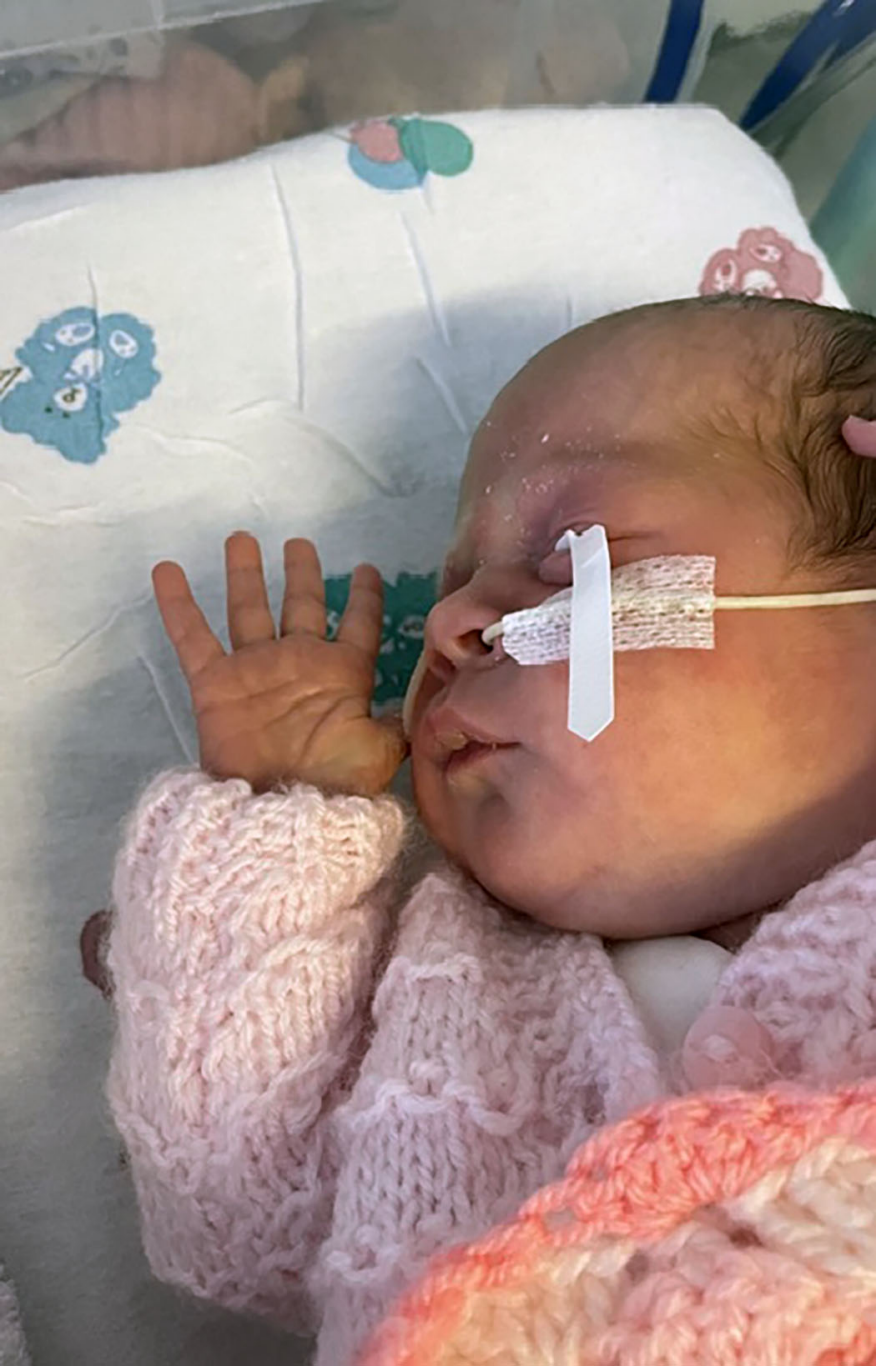
Photograph of infant having tear sample collected.

Tear sample collection was timed to coincide with routine ROP screening. Screening continued as per national guidelines and was not altered for this study. Tear samples were collected by placing strips of Schirmer's paper under the upper or lower eyelids (see Fig. 1). Proxymetacaine, cyclopentolate, and phenylephrine eye drops were administered to all infants for ROP screening as per local protocol: Schirmer strips were placed before administration of drops in some infants, and afterward in others, and the order was noted for qualitative observation of comfort and tear quantity. If instilled before tear sampling, proxymetacaine was instilled immediately before, whereas cyclopentolate and phenylephrine were instilled 0.5 to 1 hours before. Samples were collected from one or from both eyes. The number of millimeters of tear wetting was measured from the notch on the Schirmer's paper with a nominal target collection duration of 5 minutes, lengthened or shortened depending on factors such as infant's care and degree of wetting. To assess ease of sampling, the number of staff required was recorded and the collector graded ease of collection from one to five, one being the easiest. Strips were stored in Eppendorf containers where proteins were eluted, solubilized, and reduced directly from the Schirmer's paper strips into SDT buffer (4% w/v SDS, 100 mM Tris-HCl pH 7.6, and 100 mM DTT). These were then frozen immediately at −20 degrees Celsius. Samples from both eyes of any infant were combined in the container.

The filter-aided sample preparation method^18^ (alkylating using iodoacetamide) was used for trypsin digestion and dried down completely. Peptides were then resuspended in 100 mM TEAB before being tandem mass tagging (TMT) labeled, as per the manufacturer's guidelines for the 6-plex TMT reagent kits (Thermo Scientific). Peptides from patients 1 to 6 were tagged with TMT^6^-126 to 131 labels of one kit and peptides from patients 7 to 12 were tagged with TMT^6^-126 to 131 labels of a second kit. After labeling, peptides from patients 1 to 6 and patients 7 to 12 were mixed together as TMT batches 1 and 2 (1 ug total protein per TMT batch), respectively, before drying down completely and freezing at −20 degrees Celsius until MS analysis.

Dried peptides residues were solubilized in 20 µL 5% acetonitrile with 0.5% formic acid using the auto-sampler of a nanoflow ultra-high performance liquid chromatography (uHPLC) system (RSLCnano; Thermo Scientific, Waltham, MA, USA). Online detection of peptide ions was by electrospray ionization (ESI) MS with an Orbitrap Fusion MS (Thermo Scientific, Waltham, MA, USA). Ionization of liquid chromatography (LC) eluent was performed by interfacing the LC coupling device to an Triversa NanoMate (Advion Bioscience, Ithaca, NY, USA) with an electrospray voltage of 1.7 kV. An injection volume of 5 µL of the reconstituted protein digest was desalted and concentrated for 10 minutes on trap column (0.3 × 5 mm) using a flow rate of 25 µL/min with 1% acetonitrile with 0.1% formic acid. Peptide separation was performed on a Pepmap C18 reversed phase column (50 cm × 75 µm, particle size 2 µm, and pore size 100 Å; Thermo Scientific, Waltham, MA, USA) using a solvent gradient at a fixed solvent flow rate of 0.3 mL/min for the analytical column. The solvent composition was (A) 0.1% formic acid in water, and (B) 0.08% formic acid in 80% acetonitrile 20% water. The solvent gradient was 4% B for 1.5 minutes, 4 to 60% for 178.5 minutes, and 60 to 99% for 15 minutes, held at 99% for 5 minutes. A further 10 minutes at initial conditions for column re-equilibration was used before the next injection.

The Orbitrap Fusion acquires a high-resolution precursor scan at 120,000 reversed phase (RP; over a mass range of mass to charge ratio [m/z] 400–1600) followed by top speed (2.5 seconds) collision induced dissociation (CID) fragmentation (35%) and detection of the top precursor ions from the MS scan in the linear ion trap using turbo scan speed. Triple stage mass spectrometry-based approach (MS3) higher-energy collisional dissociation (HCD; 55%) is performed on the top ions from the double stage MS approach (MS2) CID scan, with up to 10 Synchronous Precursor Selection (SPS) scans isolated with the precursor ion and any TMT loss ions excluded from the selection. Orbitrap detection of the TMT quantitation label from the MS3 fragmentation is acquired at a resolution of 30,000 (for 6-plex)) with a mass range 100 to 500 m/z.

Maxquant version 1.6.14.0^19^ was used to search raw files with default settings for MS3 6-plex TMT reporter quantification utilizing isobaric matching between runs. The two 6-plex TMT batches were directly compared by using PSM-level normalization to generate normalization ratios for TMT reporter intensities,^20^ before being further normalized by median centering/log_2_ transformation. Statistical analysis of tear proteomes was carried out in Perseus version 1.6.14.0.^21^

## Results

Thirty-three infants were identified as eligible; parents of 17, including 2 sets of twins, were approached of whom 10 (12 infants) consented to participate (67% consent rate). There were seven male and five female infants. Ten of the 12 infants were White in keeping with local population ethnicity, and 2 were Black (Table 1). GA ranged from 25 + 6 to 31 + 1 (median = 29 + 4) weeks and birth weight ranged from 560 to 1670 g (median = 1225 g). No infant was small for gestational age (≤9th percentile at birth). Of the 2 sets of twins, infants 3 and 4 were dichorionic diamniotic (likely genetically nonidentical) and infants 7 and 8 were monochorionic diamniotic (genetically identical; see Table 1). Medical histories and G-ROP scores (number of positive criteria of a possible 6) are noted in Table 1. No infant had hydrocephalus. One infant (# 9) developed ROP (stage 2 zone 2) which resolved without treatment.

**Table 1.** Patient Demographics

Baby No.	Sex	Gestational Age, Wk	Postnatal Age, d at Collection	Postmenstrual Age at Sample Collection, Wk	Birth Weight, Grams	Weight at Sample Collection +/− 1 d (Percentile)	No. +ve G-ROP Criteria	Eye	Drops Prior to Schirmer	ROP?	Medical History
1	Male	25 + 6	56	33 + 6	1160	2838 (94.7)	1	Left	Nil	No	Respiratory distress, sepsis, jaundice
2	Male	29 + 0	65	38 + 2	1400	2836 (17)	1	Right	Nil	No	Patent ductus arteriosus
3 DCDA	Male	30 + 0	21	33 + 0	1640	1912 (41)	0	Right	Nil	No	Respiratory distress, craniosynostosis
4 DCDA	Male	30 + 0	27	33 + 6	1670	2428 (64.8)	0	Left	Cyclo pheny proxy	No	Craniosynostosis
5	Male	25 + 6	55	33 + 5	840	1818 (56.7)	4	Right	Proxy	No	Acquired cytomegalovirus (CMV)
								Left	Nil	No	
6	Female	31 + 1	29	35 + 2	1260	2158 (22.6)	0	RightLeft	Proxy cyclo phenyProxy cyclo pheny	NoNo	Respiratory distress, jaundice, ventricular septal defect
7 MCDA	Female	29 + 4	19	32 + 2	1190	1370 (8.9)	0	Right	Nil	No	Jaundice, respiratory distress
								Left	Proxy	No	
8 MCDA	Female	29 + 4	19	32 + 2	980	1244 (3.9)	1	Right	Nil	No	Jaundice, respiratory distress
								Left	Proxy	No	
9	Female	23 + 0	45	29 + 3	560	770 (1.9)	5	RightLeft	ProxyProxy	Yes	Chorioamnionitis, patent ductus arteriosus
10	Male	30 + 2	25	33 + 6	1510	1732 (8.2)	0	Left	Proxy	No	Respiratory distress, jaundice
11	Female	29 + 4	31	34 + 0	1560	2306 (62.9)	0	Right	Nil	No	Respiratory distress, jaundice
12	Male	27 + 0	66	36 + 3	1010	2070 (5)	3	Right	Nil	No	Respiratory distress, jaundice, group B streptococcus sepsis

If instilled, proxymetacaine (proxy) was instilled immediately before and cyclopentolate (cyclo) and phenylephrine (pheny) was instilled 30 minutes to 1 hour before. There was one set of monochorionic diamniotic (MCDA) twins and one set of dichorionic diamniotic (DCDA) twins.

At tear collection, PNA ranged from 19 to 66 days (median = 30.5 days) and PMA ranged from 29 + 3 to 38 + 2 weeks (median = 33 + 6 weeks). Weight at tear collection (+/− 1 day) ranged from 770 to 2838 g (median = 1991 g).

A total of 17 tear samples were collected: 7 infants had 1 eye sampled, and 5 infants had both eyes sampled (see Table 1). Duration of tear collection on Schirmer strips was 3 to 20 minutes (median = 5 minutes). Tear quantity collected (12 samples) measured 5 to 21 mm (median = 9.5 mm). Five samples were judged to be approximately 18 mm but were not formally measured: these were excluded from the median calculation. Of the 12 measured tear samples, the rate of wetting ranged from 0.7 to 7 mm/min. Staff required for sampling ranged from one to two (mean = 1.08). Ease of collection ranged from one to two (mean = 1.18).

Three infants had bilateral tear samples collected after receiving proxymetacaine in one eye but not the other. In two of these infants, the volume of tear collection over the same time period was 5 to 6 mm greater from the eye with proxymetacaine. In the other infant, the eye with proxymetacaine took 3 minutes longer to produce the same volume of tears as the non-instilled eye. Eyes with cyclopentolate and phenylephrine installation prior to tear collection appeared to have the slowest collection and lowest volumes: however, formal measurements were available for only three eyes from two infants. Infants seemed similarly comfortable with or without proxymetacaine prior to tear collection.

**Figure 2.**
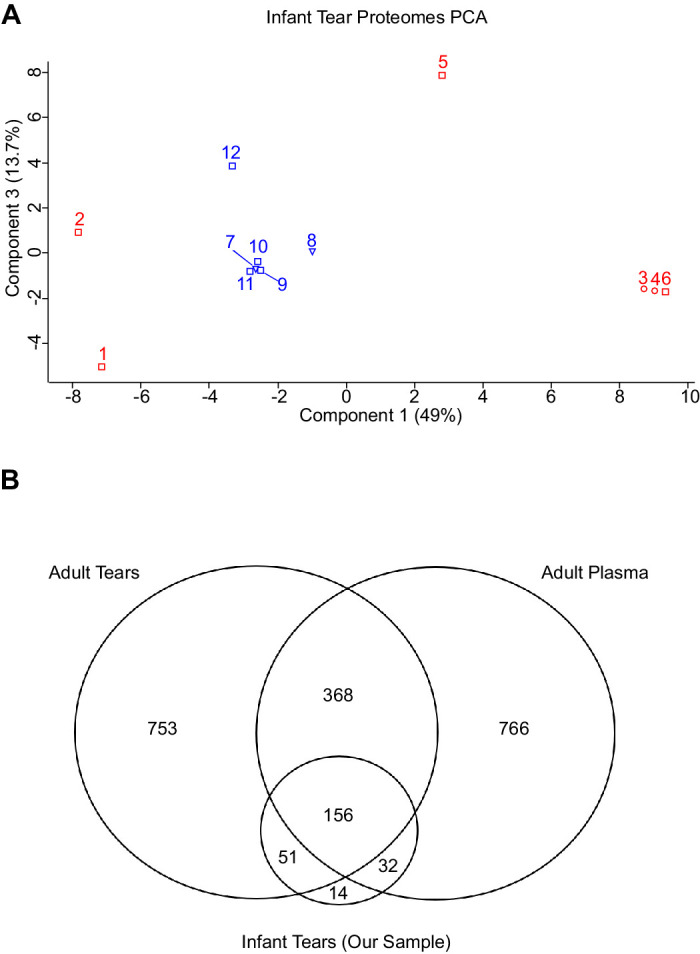
Principal component analysis was carried out on the normalized tear protein data in Supplementary Table S2, with the components (% variation between the samples) plotted on the x and y axis. (**A**) Each square representing a single patient is colored by TMT batch (*red* for batch 1 and *blue* for batch 2) with DCDA (infants 3 and 4) and MCDA (infants 7 and 8) sets of twins represented by ○ and ▼ instead, respectively. Note the close proximity of the twins, indicating significant similarity of their proteomes to their sibling. (**B**) Venn diagram showing the overlaps between quantified proteins infant tears (our sample) and published adult tear and adult plasma proteomes. Specific overlapping proteins are listed in Supplementary Table S3.

Across the 12 infants’ tear samples, 701 proteins were identified (Supplementary Table S1). Due to the high dynamic range of body fluid proteins and the difficulty in quantifying the same ions across different MS injections using data dependent acquisition,^22^ only 261 proteins were quantified in 8 of 12 infants’ tear samples and used for subsequent comparative analysis (Supplementary Table S2). Principal component analysis of the tear proteomes revealed no TMT batch effect, suggesting normalization was successful. Both sets of twins in the cohort showed exceptionally similar proteomes (minimal separation across components 1 and 2 representing 65.1% of the variability) as would be expected for genetically similar individuals (Fig. 2A).

Known tear proteins lysosome C, lipocalin-1, proline-rich protein 4, and lactotransferrin^23^ were detected as the first, second, third, and fifth most intense proteins, respectively, confirming samples did indeed include tear fluid. As serum albumin was the sixth most intense protein detected, infant tear proteomes were compared with much deeper adult plasma^24^ and adult tear^25^ proteomes. Infant tear samples had more in common with adult tears than with adult plasma (Fig. 2B), further confirmation that the samples collected represented tears. The proteins that overlap between these datasets are listed in Supplementary Table S3. VEGF was not found in our samples.

**Figure 3.**
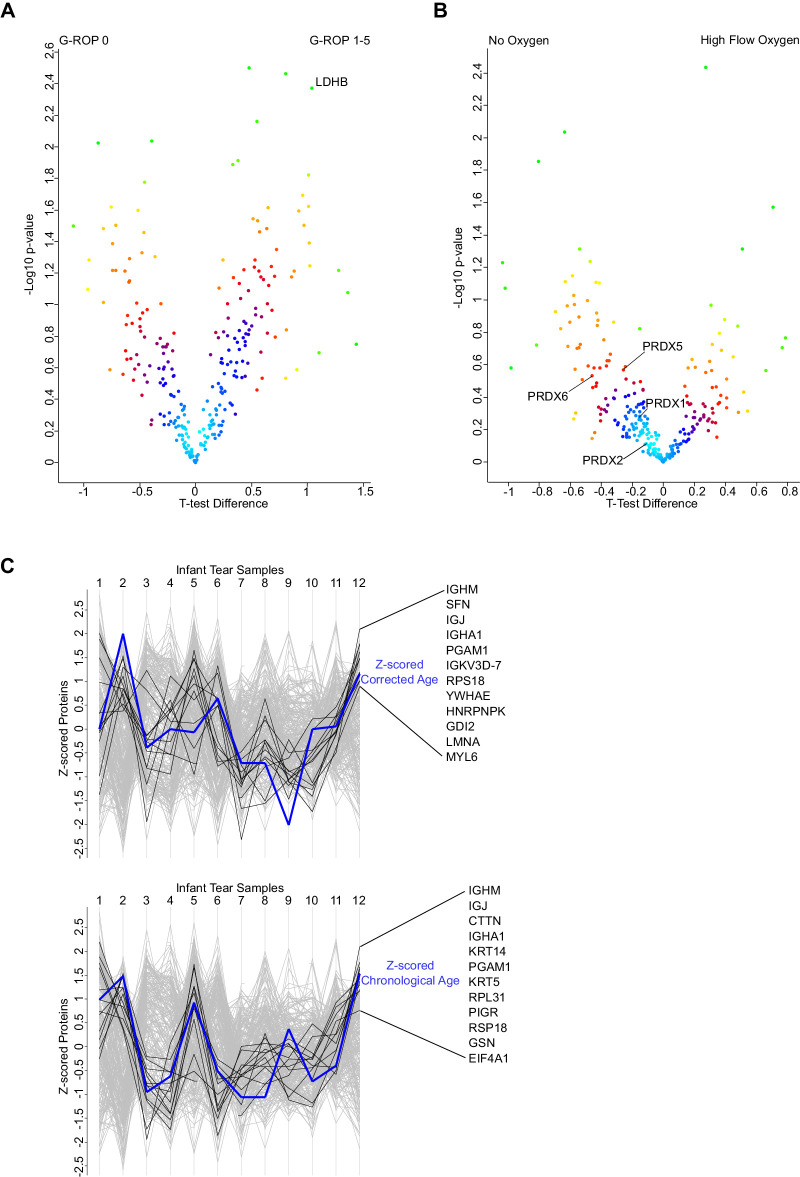
Infant tear sample proteomic results were divided into no risk and risk of ROP groups based on G-ROP criteria (G-ROP 0 vs. 1–5), and a randomized *t*-test (significance analysis of microarrays method) was carried out to identify significant differences between the two groups. (**A**) A volcano plot was generated showing the results of the statistical test – with *t*-test difference or log_2_ (fold change) plotted against -log10 (*P* value) and colored using the density estimation function in Perseus. An increased concentration of LDH was found in those with a higher risk of ROP, with a *P* value of 0.004. The top 5 most significant changes are listed in Table 2. (**B**) Infant tear sample proteomic results were divided into two groups: those on high flow supplemental oxygen versus those who were not, and a randomized *t*-test (significance analysis of microarrays method) was carried out to identify significant differences between the two groups. A volcano plot was generated showing the results of the statistical test – with *t-*test difference or log_2_ (fold change) plotted against -log10 (*P* value) and colored using the density estimation function in Perseus with all detected PRDXs (peroxiredoxins) indicated. This demonstrated that all peroxiredoxin proteins had a higher concentration in those without oxygen use, which is interesting based on their function as antioxidant enzymes. (**C**) Infant tear proteins quantifications as well as infant PMA/PNA were *z*-scored before being profile plotted. Protein *z*-scores were ranked against the age z-scores and ranked by Pearson correlation. Fisher's exact test was carried out with the highest ranking proteins (labeled) to identify enrichment of the GOCC immunoglobulin complex as a significant enrichment.

Tear samples were compared across two subgroups of infants: low risk (G-ROP score of 0, *n* = 6) versus higher risk of ROP (G-ROP score 1–5, *n* = 6). An increase in lactate dehydrogenase B (LDH-B) chain was seen in infants with a higher risk of ROP (Fig. 3A). The top 5 most statistically significant proteins with their intensities for each infant are found in Table 2.

**Table 2.** Infant Tear Sample Proteomic Results Were Divided into no Risk and Risk of ROP Groups Based on G-ROP Criteria (G-ROP 0 vs. 1-5), and a Randomized *T*-Test (Significance Analysis of Microarrays Method) was Carried out to Identify Significant Differences Between the Two Groups

	Normalized Log_2_ TMT Intensity															
Patient	3	4	6	7	10	11	1	2	8	12	5	9				
G-ROP	0	0	0	0	0	0	1	1	1	3	4	5	Average Log_2_ TMT Intensity G-ROP 0	Average Log_2_ TMT Intensity G-ROP 1-5	Fold Change (G-ROP 1-5)/(G-ROP 0)	Gene Names
	23.0536	23.0039	22.8918	22.4431	22.188	22.7967	23.5248	24.5432	22.8602	25.1032	23.9539	22.4699	22.730	23.743	**2.018**	IGHM
	26.4801	26.2659	26.2651	25.7369	25.7011	26.6109	27.4508	27.3394	26.9019	27.9458	27.6798	25.8179	26.177	27.189	**2.018**	IGHA1
	23.6251	23.4829	23.3985	21.8178	21.9627	22.0474	21.6181	20.3558	22.1117	21.7827	21.9046	21.9874	22.722	21.627	**0.468**	GPI
	21.3826	21.3974	21.2441	22.3393	22.2175	22.6313	23.4375	23.0261	22.9273	22.9958	22.6457	22.4119	21.869	22.907	**2.054**	LDHB
	20.1948	20.1834	20.0416	20.9691	21.434	21.9971	22.5625	22.0683	21.4775	21.9568	21.3368	21.4642	20.803	21.811	**2.011**	RPL4

This table shows the results of the 5 most statistically significant proteins and their intensities for each infant. A volcano plot was also generated for all results seen in Figure 3A. As tandem mass tagging (TMT) was used to quantify and multiplex samples, the TMT intensity correlates with protein abundance. Normalized log_2_ intensity can be used to compare the amount of each protein between patient samples. The fold change values describe the ratio of averaged protein intensity between the two G-ROP patient groups, with a value of >1 indicating increased intensity of proteins in the G-ROP 1 to 5 group relative to those with G-ROP 0.

Two subgroups were compared based on oxygen use at time of sample collection. Infants on high flow oxygen at collection (infants 2–5 and 7–9, *n* = 7) were compared with those not on oxygen on the day of tear collection or during the preceding week (infants 1, 6, 10, and 12, *n* = 4). Infant 11 was receiving low flow oxygen via nasal cannula on the day prior to collection but not on the day of collection and was excluded. Unsurprisingly, infants on high flow oxygen were less mature (33 + 2 vs. 34 + 6 weeks’ PMA). No individual proteins were found to differ significantly between groups; however, there was a trend for all the detected peroxiredoxin proteins (PRDXs 1, 2, 5, and 6) to be higher in tears of non-oxygen dependent infants (Fig. 3B).

Neither PMA nor PNA correlated significantly with any single protein. However, there was a significant enrichment with both PMA and PNA (21.917, false discovery rate [FDR] = 0.0149) for the gene ontology category immunoglobulin complex (GO = 0071745) among the closest correlating proteins by distance, such as proteins related to IgA and IgM (Fig. 3C). No significant proteins or enrichments were correlated with infant weight percentile on the day of collection.

## Discussion

Tear samples were collected with relative ease from a small cohort of preterm infants at risk of ROP. Highly similar proteomes in two sets of twins, plausible intensities of known tear proteins and a greater similarity to adult tears than to adult plasma proteomes, gave confidence that the samples represented preterm infant tears. Schirmer's strips could be left in place for up to 20 minutes without apparent infant distress and collected tear samples of 5 to 21 mm (approximately 4–14 µL^22^), adequate for analysis and superior in volume to studies using a cellulose rod for up to 2 minutes.^23^ Topical dilating drops (cyclopentolate and phenylephrine) may have reduced tear production: cyclopentolate is an anticholinergic and reduces tear quantity in animals.^24^ Proxymetacaine drops were associated with increased tear production in 2 out of 3 cases, perhaps by adding directly to the sample volume: each drop is 25 to 69 µL^25^ and although most of this overflows and is wiped away in neonates, some may remain in the fornix as tear drainage from nasolacrimal ducts may take a year or longer to develop.^26^ Based on our experience, ≤5 minutes is generally sufficient for tear sample collection without dilating or topical anesthetic drops.

VEGF was not found in our samples: deeper proteomic analysis may aid detection between samples. This would be useful because lower VEGF and angiogenin were found in tear samples of infants with regressing ROP compared to those that were progressing.^8^

Higher LDH-B concentration was found in infants at higher risk of ROP as calculated by the G-ROP score. The G-ROP criteria has been validated in a retrospective study of 7483 infants in North America demonstrating 100% sensitivity in detecting treatment-warranted ROP, an improvement from US criteria based on body weight and GA alone, whereas reducing the number of babies that required screening significantly.^26^

LDH catalyzes conversion of lactate to pyruvate in the anaerobic metabolic pathway and has clinical significance as a marker for tissue damage: elevated LDH has clinical significance in conditions such as myocardial infarctions and megaloblastic anemia.^29^ LDH-B mRNA mirrors oxygen levels in rat and chick retinal cells exposed to hypoxia and subsequent reperfusion, with the reverse picture for LDH-A.^30^ It may be that a similar process occurs in the preterm human neonate: healthcare laboratories already have workflows for LDH blood testing which could be adapted for tear testing, if further studies validate the diagnostic relevance of LDH in infant tears, to provide an accessible, novel method of ROP screening.

All four PRDX proteins identified tended to have lower concentrations in those infants still requiring supplemental oxygen at the time of testing, albeit without statistical significance. Peroxiredoxins are a family of enzymes which reduce peroxides and scavenge reactive oxygen species. Oxidative stress can be triggered by any imbalance in oxygen levels relative to the local tissue's normal state, such as poorly functional, chaotically organized vasculature (often seen in vessel overgrowth), or from changes in oxygen supply (antenatal air exposure or oxygen therapy).^27^ The changes in peroxiredoxin expression observed here suggest a deeper change in oxidative stress and warrant further investigation into oxidation of tear proteins themselves or other markers of ROP.

There was a statistically significant increase in immunoglobulin concentrations (including IgA and IgM) with increasing postnatal as well as maturational age. IgA is one of the most common proteins found in tears^23^; its increased abundance with PMA may represent an increase in immune response development whereas its increased abundance with PNA likely follows exposure to microbes. Because less mature infants in this study also tended to be younger, it is not possible from this small cohort to untangle the effects of maturational age and PNA in increasing immunoglobulin concentrations. This may not be significant in the pathophysiology of ROP, however, it does demonstrate maturation of the tear proteome and validation of our technique.

The study had several limitations, notably its small sample size. We have, however, demonstrated that collecting tear samples for proteomic analyses in preterm infants at risk of ROP is both feasible and well tolerated. Recording in a future study whether the infants’ eyes remain open or closed throughout collection would be helpful in exploring whether this affects tear protein content.^28^ Useful practical observations regarding methods can inform future study design.

A single 3-hour LC-MS gradient was used to analyze the tear proteome; however, as understood from other studies, there are thousands of proteins found in tears that have deeper proteomes. More subjects and deeper proteomic analysis should therefore provide more mechanistic insights into ROP. Proteins were compared between the two 6-plex testing batches using software to correct for between batch differences; however, comparison within the same plex plate is more reliable.

Identification of tear proteins predictive of the development of ROP would be a useful adjunct to ROP screening, with potential to reduce the number of infants requiring screening by clinical examination and/or identify infants at highest risk who require more frequent screening or earlier treatment. This could be by use of a lateral flow test. Better understanding of tear proteomics might also enable further exploration of ocular development and the pathogenesis of ROP as well as targeted treatments.

## Conclusions

Tear sampling by Schirmer test strips and subsequent proteomic analysis in preterm infants is feasible. An increase in the LDH-B chain was seen in infants with a higher risk of ROP and there was an increase in immunoglobulin concentrations with increasing PMA and PNA. A larger study is being designed to investigate the potential use of tear proteomics in early identification of ROP and further understanding of ocular development and the pathogenesis of ROP. This feasibility study will enable a standardized protocol for collection and analysis to be developed, as well as enable a power calculation.

## Supplementary Material

Supplement 1

Supplement 2

Supplement 3
